# Supplementary material for: Bayesian refinement of protein structures and ensembles against SAXS data using molecular dynamics
Source: PLoS Comput Biol. 2017 Oct 18;13(10):e1005800. doi: 10.1371/journal.pcbi.1005800 (PMC5662244; doi:10.1371/journal.pcbi.1005800)
Supplement: S1 Archive — (BZ2) [file pcbi.1005800.s011.tar.bz2 › gromacs_4.62_bayesian_saxs_refi/share/html/online/trjorder.html]

trjorder


| trjorder Main Table of Contents |

**VERSION 4.6  
Sat 19 Jan 2013**

---

### Description

trjorder orders molecules according to the smallest distance
to atoms in a reference group
or on z-coordinate (with option -z).
With distance ordering, it will ask for a group of reference
atoms and a group of molecules. For each frame of the trajectory
the selected molecules will be reordered according to the shortest
distance between atom number -da in the molecule and all the
atoms in the reference group. The center of mass of the molecules can
be used instead of a reference atom by setting -da to 0.
All atoms in the trajectory are written
to the output trajectory.

trjorder can be useful for e.g. analyzing the n waters closest to a
protein.
In that case the reference group would be the protein and the group
of molecules would consist of all the water atoms. When an index group
of the first n waters is made, the ordered trajectory can be used
with any Gromacs program to analyze the n closest waters.

If the output file is a .pdb file, the distance to the reference target
will be stored in the B-factor field in order to color with e.g. Rasmol.

With option -nshell the number of molecules within a shell
of radius -r around the reference group are printed.

### Files

| option | filename | type | description |
| --- | --- | --- | --- |
| **-f** | traj.xtc | Input | Trajectory: xtc trr trj gro g96 pdb cpt |
| **-s** | topol.tpr | Input | Structure+mass(db): tpr tpb tpa gro g96 pdb |
| **-n** | index.ndx | Input, Opt. | Index file |
| **-o** | ordered.xtc | Output, Opt. | Trajectory: xtc trr trj gro g96 pdb |
| **-nshell** | nshell.xvg | Output, Opt. | xvgr/xmgr file |

### Other options

| option | type | default | description |
| --- | --- | --- | --- |
| **-[no]h** | bool | no | Print help info and quit || **-[no]version** | bool | no | Print version info and quit || **-nice** | int | 19 | Set the nicelevel || **-b** | time | 0 | First frame (ps) to read from trajectory || **-e** | time | 0 | Last frame (ps) to read from trajectory || **-dt** | time | 0 | Only use frame when t MOD dt = first time (ps) || **-xvg** | enum | xmgrace | xvg plot formatting: xmgrace, xmgr or none || **-na** | int | 3 | Number of atoms in a molecule || **-da** | int | 1 | Atom used for the distance calculation, 0 is COM || **-[no]com** | bool | no | Use the distance to the center of mass of the reference group || **-r** | real | 0 | Cutoff used for the distance calculation when computing the number of molecules in a shell around e.g. a protein || **-[no]z** | bool | no | Order molecules on z-coordinate |

---

http://www.gromacs.org  
gromacs@gromacs.org
